# Suicide in prison and after release: a 17-year national cohort study

**DOI:** 10.1007/s10654-021-00782-0

**Published:** 2021-08-24

**Authors:** Anne Bukten, Marianne Riksheim Stavseth

**Affiliations:** 1 Norwegian Centre for Addiction Research, University of Oslo, Oslo, Norway; 2 Section for Clinical Addiction Research, Oslo University Hospital, Oslo, Norway

**Keywords:** Prison, Suicide, Mortality, Criminal justice, Mental health, Release, Convictions

## Abstract

**Background:**

People in prison have an extremely high risk of suicide. The aim of this paper is to describe all suicides in the Norwegian prison population from 2000 to 2016, during and following imprisonment; to investigate the timing of suicides; and to investigate the associations between risk of suicide and types of crime.

**Methods:**

We used data from the Norwegian Prison Release study (nPRIS) including complete national register data from the Norwegian Prison Register and the Norwegian Cause of Death Register in the period 1.1.2000 to 31.12.2016, consisting of 96,856 individuals. All suicides were classified according to ICD-10 codes X60-X84. We calculated crude mortality rates (CMRs) per 100,000 person-years and used a Cox Proportional-Hazards regression model to investigate factors associated with suicide during imprisonment and after release reported as hazard ratios (HRs).

**Results:**

Suicide accounted for about 10% of all deaths in the Norwegian prison population and was the leading cause of death in prison (53% of in deaths in prison). The CMR per 100,000 person years for in-prison suicides was 133.8 (CI 100.5–167.1) and was ten times higher (CMR = 1535.0, CI 397.9–2672.2) on day one of incarceration. Suicides after release (overall CMR = 82.8, CI 100.5–167.1) also peaked on day one after release (CMR = 665.7, CI 0–1419.1). Suicide in prison was strongly associated with convictions of homicide (HR 18.2, CI 6.5–50.8) and high-security prison level (HR 15.4, CI 3.6–65.0). Suicide after release was associated with convictions of homicide (HR 3.1, CI 1.7–5.5).

**Conclusion:**

There is a high risk of suicide during the immediate first period of incarceration and after release. Convictions for severe violent crime, especially homicide, are associated with increased suicide risk, both in prison and after release.

**Supplementary Information:**

The online version contains supplementary material available at 10.1007/s10654-021-00782-0.

## Introduction

Suicide is a major public health concern. Globally, nearly 800 000 people die each year due to suicide, and suicide is the second leading cause of death among 15–29-year-olds [1]. People in prison have an extremely high risk of suicide; compared with people in the general population of the same sex and similar age, suicide rates of men are three times higher and for women, nine times higher [2]. Suicide is the single most common cause of death in prisons [3] and among the leading causes of death following release [4].

Various factors contribute to elevated suicide rates among people in prison. First, several well-established demographical and clinical risk factors are consistently overrepresented among prisoners upon arrival [3, 5–14]. In a recent review and meta-analysis, Zhong and colleagues found that the strongest clinical factors associated with suicide in prisons were suicidal ideation during the current period in prison, a history of attempted suicide, and current psychiatric diagnosis [14].

Additionally, the prison environment may contribute to an elevated risk of suicide [15], and the impact is likely magnified among people with individual risks. Having no social visits [14, 16] and factors such as isolation and single cell have been found to be associated with suicide in prisoners [8, 13, 17].

Criminological factors are also associated with suicide in prisoners, and studies have found elevated suicide risk among people imprisoned for severe violent offences [13, 18], in particular homicide [14, 16] and according to remand status and serving a life- sentence [14]. In sum, factors associated with prisoner suicide include a range of demographical, clinical, environmental and criminological factors [3, 19].

Different time periods related to imprisonment and release have been associated with higher risk of suicide. The first weeks of imprisonment are associated with higher risks [20], especially among young males on remand [21]. Also, the period following release represents a vulnerable transition, involving a high risk of self-harm and suicide [22–24] and several studies have demonstrated high rates of suicides in the immediate period post release [7, 25, 26].

Prison suicide is a global problem, but its extent varies between countries. International studies comparing suicide rates have found that the Nordic countries have the highest prison suicide rates globally [2, 27]. In a recent meta-analysis investigating prion suicides in 24 counties, Norway peaked with 180 suicides per 100 000 prisoners and as a country with a substantially increased risk of suicide amongst prisoners compared to the general population [2].

Suicides are preventable with timely, evidence-based, and often low-cost interventions [1]. However, for national responses to be effective, more information is needed to identify high-risk periods for suicide and modifiable risk and protective factors. The aim of this study is to: 1) describe all suicides in the Norwegian prison population from 2000 to 2016, in prison and after release, 2) to investigate the timing of suicides with regard to admission to prison and prison release, and 3) to investigate the association between types of crime and risk of suicide and criminal offences.

## Material and methods

### Setting

Norway is characterized by having low rates of imprisonment that aim at rehabilitation, and in which universal health care, including drug treatment, is provided. In 2020, the prison population rate per 100 000 of the national population was 49 in Norway, compared to 639 in the US and 132 in the UK [28].

In 2019, Norwegian prisons had the capacity of 3 550 prison beds and the mean number of inmates were 3 218 [29]. Prison beds are spread over 49 prison units, a form of prison organization that allows most prisoners to preserve geographical closeness to friends and family. Norwegian prisons vary in size; the largest prison has a capacity of 400 people, while the smallest has only 15.

All prisons are publicly funded and are categorized into high-security (almost two-thirds of prisons), low-security, or transitional housing units. Ideally, inmates begin serving their sentences in high-security prisons before being transferred to a prison with low security and subsequently to transitional housing units. However, most inmates are released from high-security units.

Of all releases from prison during 2018, about 20% of the inmates were released after 30 days or less and about 85% were released within one year. Women constitute a minority in Norwegian prisons, with an annual proportion of approximately 6% [30].

### Design and population

The design is a retrospective cohort study. We used data from the Norwegian Prison Release study (nPRIS). This cohort study includes all people imprisoned in Norway over a 17-year period (January 1^st^, 2000 until December 31^st^, 2016) collected from the Norwegian Prison Registry, including 114 745 individuals contributing 187 046 releases. The data were linked to the Norwegian Cause of Death Register using a unique 11-digit Personal Identification Number (PIN) given to all residents.

A total of 19 140 individuals were excluded from the study cohort, resulting in a study population of 96 856 individuals. The majority (96.8%) of these exclusions were due to not having Norwegian PINs, which is a precondition for linking to registry data.

### Data sources

The Norwegian Prison Registry (NPR) serves administrative and statistical purposes, and includes personal data on all persons imprisoned in Norway, including age, gender, convictions, and sentences [31]. The registry also includes date of admission and date of release, both for sentences served and time spent on remand. This includes a code describing the release circumstances, which differentiates between transfers to hospitals, rehabilitation institutions, deaths in prison and actual prison release. If a person is hospitalized during imprisonment, this will not be categorized as a release.

The Norwegian Cause of Death Register (NDR) includes complete death certificates reported by medical doctors after examination of the deceased. Death certificates are collected by the NDR at the Norwegian Institute of Public Health. All deaths are coded using the International Classification of Diseases, 10th revision (ICD-10) [32]. The NDR includes information about the underlying cause of death (the disease or injury which initiated the chain of morbid events leading directly to death) and immediate causes of death (the terminal event or complication present at the time of death) [33]. Information about where the person was found dead and the actual date of death is also included. The coverage and the completeness of the NDR is high; it comprises all Norwegian residents and include medical information on more than 98% of all deaths [33].

### Measures

Causes of death were categorized as either natural or unnatural; natural causes were defined as ICD-10 chapters A through Q, while unnatural causes were defined as ICD-10 chapters V through Y.

In-prison suicides are defined by suicide-deaths occurring in prison. Persons who initiated “the suicide act” in prison, but died outside of prison, e.g. in the hospital, are not included. Suicides occurring any time after release are defined as ‘after release’. We considered only suicides with fatal outcome, coded X60 through X84 according to the ICD-10.

Method of suicide were categorised as: Intentional self-poisoning (X60-69), Intentional self-harm by hanging, strangulation and suffocation (X70), Intentional self-harm by firearm (X72-74), and “Other or unknown method” (X76-77, X79, X81-84).

In the study cohort, most imprisonments included multiple convictions. “Main conviction” was defined as the most severe conviction. Types of crime were classified into ten groups. Nine were based on Statistics Norway’s official crime statistics. In addition we included a separate group for homicide: (1) Property theft, (2) Other offences for profit, (3) Criminal damage, (4) Drug related offences, (5) Public order and integrity violations, (6) Sexual offences, (7) Traffic offences, (8) Violence and maltreatment, (9) Other offences [34] and (10) Homicide.

When calculating the CMRs for main convictions and Cox Proportional-Hazards models for suicides, the nine groups were collapsed into four groups due to small number of events per group: traffic offences, public order and integrity violations, criminal damage, property theft, other offences for profit and other offences were combined into one group named ‘Public order and offences for profit’. Sexual offences and violence and maltreatment were combined into a group named ‘Sex and violence’. Drug-related offences and homicide were kept as separate groups.

Prison security level at release was categorized into high security, low security and other. The category “other” includes transfers to hospital etc.

### Time at risk

We defined two follow-up periods. *In prison:* The period between the first day of imprisonment and death, between first day of imprisonment and release, or between first day of imprisonment and the end of observation (December 31^st^, 2016). *After prison:* The period between release and death, from release to another imprisonment, or from release to the end of the observation period (December 31^st^, 2016). For the person contributing with multiple incarcerations and releases, all periods were included in the analysis.

101 deaths occurred during sentence but outside of prison (at a hospital etc.). Consequently, the date of release was set to the date of death and these deaths were excluded from the time-to-event analysis.

### Statistical analysis

Descriptive statistics were conducted using IBM SPSS 26. Crude mortality rates (CMRs) and 95% confidence intervals were calculated as number of deaths per 100 000 person years (PY) [35].

To simultaneously evaluate the effect of several factors on timing of suicides we fitted Cox Proportional-Hazards models using R version 4.0.1. Separate models were fitted for in-prison and after release suicides. In the multivariate model for in prison suicides, age at incarceration (continuous), gender (binary; man/woman), type of imprisonment (binary; pre-trial detention/sentenced), security level (nominal: high, low, other), main conviction (nominal; see above) and first incarceration (binary; yes/no) were adjusted for. In the multivariate model for suicides after release we included age at release (continuous), gender (binary; man/woman), main conviction (nominal; see above), and the number of previous releases (continuous).

The proportional-hazards assumption was tested based on the Schoenfeld residuals [36] and our hypothesis did not violate the proportional-hazards assumption (data not shown). The coefficients were interpreted in terms of incidence hazard ratios (HR) with 95% confidence intervals. Separate models were fitted to suicides in prison and after release.

## Results

Our cohort consisted of 96 856 individuals (10.3% females) contributing with 166 767 incarcerations. Median age at first incarceration was 31 (IQR: 23–41). 68.5% had one incarceration, while about 15% of the cohort had served 3 or more prison sentences (Table 1). Most imprisonments (75.4%) were between 0–3 months, while 6.2% were 12 months or more. In total, 8 053 deaths were recorded in the cohort; of those, 42.5% were characterised as unnatural causes, including 811 (10.1%) suicides (Table 1).

**Table 1:** Demographic characteristic of the study population (*n* = 96 856) observed from 2000–2016

Individuals	96,856	100% (%)
*Age at first incarceration*^a^		
< 20	6598	6.8
20–29	38,648	39.9
30–39	23,743	24.5
40–49	16,334	16.9
50–59	8316	8.6
60–69	2713	2.8
> 70	462	0.5
*Gender*^b^		
Female	9962	10.3
Male	86,875	89.7
*Number of incarcerations*		
1	66,350	68.5
2	15,746	16.3
3	6305	6.5
4	3257	3.4
5 +	5198	5.4
*Total deaths*^*c*^	8 053	100
Natural causes	4,093	50.8
Unnatural cause	3,425	42.5
*Suicide*	*811*	*10.1*
Unknown/no cause given	535	6.6

^a^42 persons missing age information

^b^19 persons missing gender information

^c^Natural cause: ICD-10 chapters A-Q, unnatural cause: ICD-10 chapters V–Y

In total, 62 suicides occurred in prison and suicide was the leading cause of death in prison (53% of in deaths in prison). Most suicides were committed by men (93.5%), and most often (87.1%) by means of hanging, strangulation and suffocation (X70). More than one fifth (21%) of all in-prison suicides occurred within the first week and almost four fifths (77.4%) were committed on pre-trail detention (Table 2).

**Table 2:** Characteristics of persons who committed suicide in prison (*n* = 62) and after release (*n* = 749), 2000–2016

	In prison		After release		Total	
	*n*	%	*n*	%	*n*	%
*Age at death*						
< 20	1	1.6	7	0.9	8	1.0
20–29	19	30.6	195	26.0	214	26.4
30–39	23	37.1	230	30.7	253	31.2
40–49	11	17.7	191	25.5	202	24.9
50–59	6	9.7	95	12.7	101	12.5
60–69	1	1.6	26	3.5	27	3.3
> 70	1	1.6	5	0.7	6	0.7
*Gender*						
Female	4	6.5	66	8.8	70	8.6
Male	58	93.5	683	91.2	741	91.4
*Type of suicide*						
Intentional self-poisoning (X60-69)	3	4.8	216	28.8	219	27.0
Intentional self-harm by hanging, strangulation and suffocation (X70)	54	87.1	332	44.0	386	48.0
Intentional self-harm by firearm (X72-74)	0	0.0	82	10.9	82	10.1
Other (X71, X75-X84)	4	6.5	119	15.9	123	15.2
*Timing (after incarceration/after release)*						
On 1st day	7	11.3	8	1.1		
Within 1st week	6	9.7	11	1.5		
Within 2nd week	7	11.3	13	1.7		
Within 1st month	5	8.1	14	1.9		
Within 1st year	31	50.0	130	17.4		
*Type of imprisonment*						
Pre-trial detention	48	77.4				
Sentenced	14	22.6				
*Prison security level*						
High security	53	85.5				
Low security	2	3.2				
Other	7	11.3				

In total, 749 suicides occurred after prison (9.4 of all deaths after release). Of all suicides occurring after release (*n* = 749), 91.2% were committed by men. Common methods were hanging (44.0%), self-poisoning (28.8%) and intentional firearm (10.9%) (Table 2).

Almost half of the people committing suicide in prison were convicted for severe violent crime, including violence and maltreatment (33.9%) or a sexual offence (12.9%). About one quarter were convicted for drug and alcohol offences (25.8%). Half of suicides (50.0%) were committed by people serving their first prison sentence, while one quarter (24.2%) had four or more prior incarcerations (Table 3).

**Table 3:** Criminal characteristics of persons who commit suicide in prison (n = 62) and after release (n = 749), 2000–2016

	In prison		After release		General prison population	
	*n* = 62	%	*n* = 749	%	*n* = 96 856	%
*Main conviction (last sentence)*^a^						
Traffic offences	0	0.0	59	7.9	8799	9.1
Public order and integrity violations	0	0.0	27	3.6	3423	3.5
Drug and alcohol offences	16	25.8	259	34.6	31,936	33.0
Sexual offences	8	12.9	16	2.1	4808	5.0
Violence and maltreatment	21	33.9	165	22.0	20,491	21.2
*Homicide—incl. attempt*	*10*	*16.1*	*12*	*1.6*	*712*	*0.7*
Criminal damage	0	0.0	12	1.6	610	0.6
Other offences for profit	2	3.2	39	5.2	10,181	10.5
Property theft	4	6.5	68	9.1	5342	5.5
Other offences	1	1.6	19	2.5	2576	2.7
*Missing*	*0*	*0.0*	*73*	*9.7*	*7978*	*8.2*
*Number of incarcerations*^b^						
1	31	50.0	478	63.8	66,350	68.5
2	8	12.9	134	17.9	15,746	16.3
3	6	9.7	70	9.3	6305	6.5
4	2	3.2	25	3.3	3257	3.4
5 +	15	24.2	42	5.6	5198	5.4

^a^Main conviction defined as the most serious type of crime in the last observed sentence in the study period

^b^Number of incarcerations during the study period

People committing suicide after release were most often convicted for drug and alcohol offences (34.6%) and violence and maltreatment (22.0%). About 70% of all committing suicide after release had only one prior prison sentence, while one fifth (18.3%) had three or more previous incarcerations (Table 3).

**Fig. 1:**
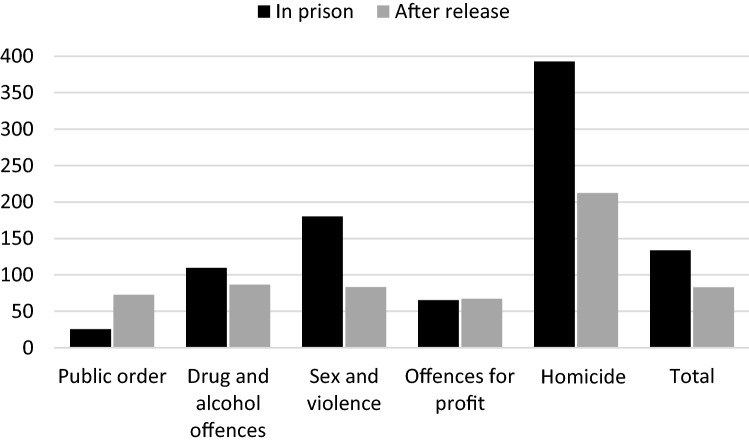
Crude mortality rates per main conviction, by in-prison suicides (n=62) and suicides after release (n=749), 2000–2016

The average crude mortality rate (CMR) in-prison suicide was 133.8 per 100 000 prisoners. When stratifying by main conviction during the last prison sentence, the CMR for suicide among people convicted of homicide was 393.0 180.0 for sexual and violence offences and 109.8 for drug and alcohol offences (Fig. 1). For details, including confidence intervals, see Supplementary Table 1.

The average crude mortality rate (CMR) after-prison suicide was 82.8 per 100 000 prisoners. When stratifying by main conviction during the last prison sentence, the CMR for suicide among people convicted of homicide was 212.4, 86.6 for drug and alcohol offences and 83.5 for sexual and violence offences (Fig. 1). For details, including confidence intervals, see Supplementary Table 1.

**Fig. 2:**
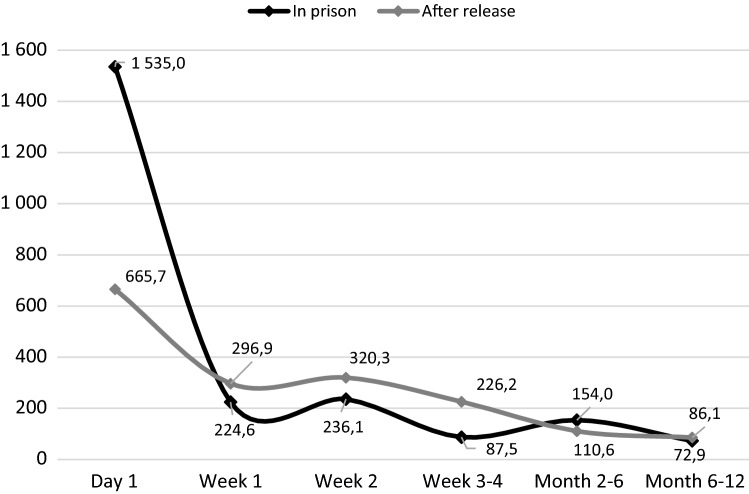
Crude mortality rates for different time units after imprisonment or after release, by in-prison suicides (n=62) and suicides after release (n=749), 2000–2016

When stratifying the time-period after incarceration and release, the CMR after incarceration was 1535.0 at day 1, 224.6 at week 1, and 154.0 at months 2–6. The CMR after release was 665.7 at day 1, 296.9 at week 1 and 110.6 at months 2–6 (Fig. 2). For details, including confidence intervals, see Supplementary Table 2.

The adjusted Cox regression model showed that in-prison suicide was associated with age at incarceration (HR 1.03, CI 1.01–1.06), being convicted of homicide (HR 18.20, CI 6.52–50.79), sexual and violent offences (HR 4.90, CI 2.14–11.24), drug and alcohol offences (HR 4.90, CI 1.26–7.56), pre-trail detention (HR 3.34, CI 1.78–6.27) and high-security unit (HR 15.36, CI 3.63–65.03) (Table 4a).

**Table 4:** Factors associated with suicides in the prison population. Separate Cox Proportional-Hazards models with associated hazard ratios (HRs) and 95% confidence intervals (CIs) estimated for **a** in-prison suicides and **b** suicides after release

(a) In prison model	HR	95% CI	*p*-value
Age at incarceration	1.03	(1.01–1.06)	0.002
*Gender (ref: female)*			
Male	0.71	(0.30–2.30)	0.712
*Type of crime (ref: public order and offences for profit)*			
Drug and alcohol offences	4.90	(1.26–7.56)	0.014
Sex and violence	4.90	(2.14–11.24)	0.000
Homicide	18.20	(6.52–50.79)	0.000
*First incarceration (ref: yes)*			
No	0.84	(0.50–1.41)	0.512
*Type of imprisonment (ref: sentenced)*			
Pre-trial detention	3.34	(1.78–6.27)	0.000
*Prison security level (ref: low)*			
High	15.36	(3.63–65.03)	0.000
Other	5.43	(1.10–26.78)	0.038

(b) After release model			
Age at release	1.00	(1.00–1.01)	0.596
*Gender (ref: female)*			
Male	1.19	(0.89–1.58)	0.239
*Type of crime (ref: ref: public order and offences for profit)*			
Drug and alcohol offences	1.25	(1.05–1.50)	0.014
Sex and violence	1.16	(0.95–1.41)	0.135
Homicide	3.05	(1.71–5.46)	0.000
Number of releases	1.11	(1.06–1.15)	0.000

Risk of suicide after release was associated with being convicted of homicide (HR 3.05, CI 1.71–5.46), drug and alcohol offences (HR 1.25, CI 1.05–1.50) and number of prior incarcerations (HR 1.11, CI 1.06–1.15).

## Discussion

Our study reports findings from all registered suicides among the total Norwegian prison population within a 17-year period. During 2000–2016, suicide was the leading cause of death during imprisonment and among the leading causes of death after release. The mean suicide rate was 133.8 per 100 000 in prison, and 82.8 per 100 000 after release. In comparison, the annual global age-standardized suicide rate of 11.4 per 100 000 population in 2012 [1].

The immediate periods after imprisonment and after release both imposed a high risk of suicide; the suicide rate on day 1 was almost seven times higher than week 1, and about ten times higher than months 2–6. Although the suicide rates after release were lower, we also found a similar trend related to time after release; the risk of suicide on day 1 after release was more than twice as high as week 1 after release, and six times higher than 2–6 months after release.

Our results documenting a peak in suicides immediately after imprisonment are in line with prior international findings showing an increased risk during the first weeks and days in prison [20, 37]. We also found an increased suicide risk among people on pre-trail detention and in high-security units supporting the existing international literature [38–41].

Although the suicide rates were lower after release, we found a doubled risk of suicide on day 1 post-release, compared to the rest of the week. With the exception of one study taking place in the northeast of Australia [24], our findings are in line with previous studies finding a peak in suicides after release [7, 20, 26]. The high overall suicide rate supports the idea that the transition to life outside prison is a period with substantially increased risk for premature death. Previous research has also suggested increased risks for overdose death during the immediate period post release [4, 24].

One main finding was the association between suicide both in prison and after release and being convicted of homicide: the risk of suicide in prison was more than 18 times higher among people convicted of homicide, adjusted for other factors. The link between violent crimes and suicide has also reported in other studies [13, 16–18]. In a recent systematic review and meta-analysis examining risk factors associated with suicide in prisoners, Zhong and colleagues found that several clinical, institutional, and criminological factors were associated with suicide among people in prison. Criminological factors included remand status, serving a life sentence, and being convicted of a violent offence, in particular homicide [14].

In line with Zhong’s and colleagues recent study [14], pre-trail detention were also an important risk factor for in-prison suicide in our cohort.

In a meta-analysis of prisoner suicide rates in 24 high-income countries in Europe, Australasia, and North America, Fazel and colleagues found that the rates of prisoner suicide were *higher* in countries where *fewer* individuals were imprisoned per 100 000 members of the general population [2]. The authors proposed an explanation for this link related to the prisoners being more selected in terms of having sentences for more serious or violent offences and more likely to be suffering from mental illnesses, and thus more vulnerable.

### Strengths and limitations

Using mandatory national registries is a major strength of the study. The datasets are linked using unique 11-digit identifiers assigned to all residents in Norway, minimizing the risk of linkage-biases. Moreover, all deaths are classified according to the most recent ICD criteria, and death-categories are reported according to individual ICD codes, minimizing the risk of information bias.

Having a national cohort followed for 17 years enables stratified analysis, which is another major strength of the study. Our study is based on a large sample, and our results advance more precise day‐by‐day understanding of risk of suicide following incarceration and release. However, suicide is a rare event, and when analysing stratified groups, such as suicides per main conviction, some groups will have small numbers. This results in higher uncertainty, reflected in wide confidence intervals.

Another limitation is the lack of demographic and socio-cultural variables in our dataset, in addition to information on mental health – factors that are associated with suicide in prisoners [8]. In Norway, one inmate per cell is most common. Sharing cells only happens occasionally and this information was thus not integrated in our dataset.

Misclassifications of causes of death may occur in registry data: it may be that some suicides might be classified as overdose deaths or accidents, causing an underestimation. However, Norwegian data has been assessed as having good validity and reliability for suicide classification [42].

Moreover, the number of suicides in prison may be somewhat underestimated due to how suicides are recorded in the databases. A person may have initiated a suicidal act in prison but dies in hospital later. Such mortalities will not be recorded as in-prison suicides in this article, as death occurred outside of prison.

## Conclusions and implications

Our study showed that the immediate period after imprisonment and after release impose a high risk of suicide, especially among people convicted of homicide.

Suicide is a serious public health problem and the World Health Organization has prioritized reducing suicides in target 3.4 of the Sustainable Development Goals [43]. According to World Health Organization guidelines, the identification of high-risk groups is crucial to effective, public health suicide prevention approaches [3]. The peak in suicide on day one of incarceration highlights the importance of high alertness towards suicidal behaviour in recently admitted prisoners, and guidelines must therefore emphasize the need for risk assessment immediately after imprisonment [3]. In addition, being on pre-trial detention and serving in a high-security unit, should serve as important markers for increased risk.

Furthermore, our results highlight the critical period following release, adding to the literature addressing the vulnerable transition from prison to society. In order to bridge this gap, comprehensive provision of health care services is necessary throughout prison and after release alongside with social re‐integration support for former prisoners.

## Supplementary Information

Below is the link to the electronic supplementary material.

Supplementary file1 (DOCX 14 kb)

Supplementary file2 (DOCX 14 kb)

## Declarations

### Data availability

This population study was based on individual-level data from The Norwegian Prison Registry (held by the Directorate of Norwegian Correctional Service) and the Norwegian Cause of Death Register (held by the Norwegian Institute of Public Health). The ethical approval of this research project does not include permission to publicly share the raw data. Qualifying researchers can apply for access to relevant data with the Norwegian Institute of Public Health [44] and the Directorate of Norwegian Correctional Service [45] upon approval from the Regional Committees for Medical and Health Research Ethics [46].
