# Supplementary material for: Suicide in prison and after release: a 17-year national cohort study
Source: Eur J Epidemiol. 2021 Aug 24;36(10):1075–83. doi: 10.1007/s10654-021-00782-0 (PMC8542551; doi:10.1007/s10654-021-00782-0)
Supplement: Supplementary file 1 — Supplementary file1 (DOCX 14 kb) [file 10654_2021_782_MOESM1_ESM.docx]

| **Supplementary table 1.** Number of suicides, person years (PYs) and crude mortality rates (CMRs) per 100 000 PY including 95% confidence intervals (CIs) per main conviction, by in-prison suicides (n=62) and suicides after release (n=749), 2000-2016. | | | | | | |
| --- | --- | --- | --- | --- | --- | --- |
| **Main conviction** | **In prison** | | | **After release** | | |
|  | Suicides | PY | CMR (95% CI) | Suicides | PY | CMR (95% CI) |
| Public order and offences for profit | 7 | 13 109 | 53.4 (13.8-93.0) | 224 | 319 731 | 70.1 (60.9-79.2) |
| Drug and alcohol offences | 16 | 14 575 | 109.8 (56-163.6) | 259 | 299 163 | 86.6 (76-97.1) |
| Sex & violence | 29 | 16 110 | 180 (114.5-245.5) | 181 | 216 777 | 83.5 (71.3-95.7) |
| Homicide | 10 | 2 545 | 393 (149.4-636.5) | 12 | 5 650 | 212.4 (92.2-332.5) |
| *Missing*^a^ | *0* | *-* | *-* | *0* | *63 009* | *115.9 (89.3-142.4)* |
| Total | 62 | 46 339 | 133.8 (100.5-167.1) | 749 | 904 331 | 82.8 (100.5-167.1) |

^a^ Imprisonments missing information about type of crime
