# Supplementary material for: Suicide in prison and after release: a 17-year national cohort study
Source: Eur J Epidemiol. 2021 Aug 24;36(10):1075–83. doi: 10.1007/s10654-021-00782-0 (PMC8542551; doi:10.1007/s10654-021-00782-0)
Supplement: Supplementary file 2 — Supplementary file2 (DOCX 14 kb) [file 10654_2021_782_MOESM2_ESM.docx]

**Supplementary table 2. Number of suicides, person years (PYs) and crude mortality rates (CMRs) including 95% confidence intervals (CIs) for different time units after imprisonment or after release, by in-prison suicides (n=62) and suicides after release (n=749), 2000-2016.**

|  | **In prison** | | | | **After release** | | | |
| --- | --- | --- | --- | --- | --- | --- | --- | --- |
| **Time period** | Suicides | PY | CMR | 95% CI | Suicides | PY | CMR | 95% CI |
| Day 1 | 7 | 456 | 1 535.0 | 397.9-2672.2 | 3 | 451 | 665.7 | 0-1419.1 |
| Week 1 | 6 | 2671 | 224.6 | 44.9-404.3 | 8 | 2 695 | 296.9 | 91.2-502.6 |
| Week 2 | 7 | 2965 | 236.1 | 61.2-410.9 | 10 | 3 123 | 320.3 | 121.8-518.7 |
| Week 3-4 | 4 | 4571 | 87.5 | 1.8-173.3 | 14 | 6 189 | 226.2 | 107.7-344.7 |
| Month 2-6 | 26 | 16880 | 154.0 | 94.8-213.2 | 65 | 58 746 | 110.6 | 83.7-137.5 |
| Month 6-12 | 6 | 8232 | 72.9 | 14.6-131.2 | 64 | 74 369 | 86.1 | 65-107.1 |
